# The Impact of Solution Ionic Strength, Hardness, and pH on the Sorption Efficiency of Polychlorinated Biphenyls in Magnetic Nanocomposite Microparticle (MNM) Gels

**DOI:** 10.3390/gels9040344

**Published:** 2023-04-18

**Authors:** Angela M. Gutierrez, Thomas D. Dziubla, J. Zach Hilt

**Affiliations:** 1 Department of Civil Engineering, University of Kentucky, Lexington, KY 40506, USA; 2 Department of Chemical and Materials Engineering, University of Kentucky, Lexington, KY 40506, USA; 3 Superfund Research Center, University of Kentucky, Lexington, KY 40506, USA

**Keywords:** polychlorinated biphenyls (PCBs), nanotechnology, nanocomposites, water quality

## Abstract

Environmental conditions of groundwater and surface water greatly vary as a function of location. Factors such as ionic strength, water hardness, and solution pH can change the physical and chemical properties of the nanocomposites used in remediation and the pollutants of interest. In this work, magnetic nanocomposite microparticle (MNM) gels are used as sorbents for remediation of PCB 126 as model organic contaminant. Three MNM systems are used: curcumin multiacrylate MNMs (CMA MNMs), quercetin multiacrylate MNMs (QMA MNMs), and polyethylene glycol-400-dimethacrylate MNMs (PEG MNMs). The effect of ionic strength, water hardness, and pH were studied on the sorption efficiency of the MNMs for PCB 126 by performing equilibrium binding studies. It is seen that the ionic strength and water hardness have a minimal effect on the MNM gel system sorption of PCB 126. However, a decrease in binding was observed when the pH increased from 6.5 to 8.5, attributed to anion-π interactions between the buffer ions in solution and the PCB molecules as well as with the aromatic rings of the MNM gel systems. Overall, the results indicate that the developed MNM gels can be used as magnetic sorbents for polychlorinated biphenyls in groundwater and surface water remediation, provided that the solution pH is controlled.

## 1. Introduction

Water conservation and quality are some of the most important global challenges humans are facing in the 21st century. Fast industrialization implies exhaustive consumption of fresh water and groundwater for agricultural, industrial, and domestic purposes [1,2]. Most of these uses have led to the contamination of water bodies with an array of pollutants. In order to mitigate the health and environmental risks associated with contamination, stringent environmental regulations have been imposed worldwide [3,4,5].

Several chemical, physical, and biological water-treatment technologies exist for the removal of organic contaminants [6,7,8,9]. Among these, adsorption processes have been extensively used to remove a wide variety of pollutants due to their large surface areas, mechanical strengths, tunable shapes and morphologies, high efficiencies, and their ease of implementation [10,11,12,13]. In recent years, the development of nanocomposite sorbent materials that combine properties of organic and inorganic materials has been of particular interest [14,15,16]. Specifically, magnetic nanocomposite microparticle (MNM) gels have been developed, wherein iron oxide nanoparticles (IO MNPs) are embedded within the composite to grant the material magnetic properties. These magnetic nanocomposite sorbents allow for a fast, easy, and cost-effective separation of the saturated sorbent from the treated solution method [17,18,19,20,21,22,23]. Furthermore, the organic component of the nanocomposite can be tailored specifically to target the sorbate of interest. Common organic components used in nanocomposites for sorption of polycyclic aromatic hydrocarbons (PAHs) contain aromatic groups that can allow for π-π interactions and hydrophobic interactions, indicating that this is the mechanism through which sorption occurs [17,20,21,22,24,25,26,27,28,29,30,31,32,33,34].

Contamination with polycyclic aromatic hydrocarbons (PAHs) is widely distributed in the environment. Because of environmental cycling, PAHs are found all over the world in groundwater, surface water, sediments, and the atmosphere. Polychlorinated biphenyls (PCBs) are a class of PAHs composed of chlorinated biphenyl complexes with varying degree of chlorination and hence varying physico-chemical properties and toxicities. In general, PCBs possess poor aqueous solubility and low volatility, which makes their environmental remediation challenging [35,36]. The U.S. Environmental Protection Agency (EPA) and the European Union (EU) have classified some PCBs as priority pollutants for monitoring and remediation purposes in natural waters [2,36]. However, due to their trace concentration in environmental waters and the complexity of environmental conditions of natural waters, the need for a remediation technique with high capacity for PCBs that is stable under environmental conditions is necessary.

Environmental conditions in groundwater and surface water can change the physical and chemical properties of the sorbent nanocomposites and the sorbate molecules. Conditions such as ionic content, water hardness, and pH vary based on the geographical location of the water body. Bedrock erosion, the presence of igneous rocks, drainage regions with alkaline earths, the presence of microbiota and microorganisms’ communities, and human influences on water sheds are some of the environmental conditions that can alter a water body’s properties. Therefore, it is of utmost importance to investigate the effect of water environment on the sorption behavior of magnetic nanocomposite materials. The main objective of this work is to evaluate the effects of different environmental factors on the sorption capacity for PCB 126, as model contaminant on the MNM gels previously developed by our group [22]. Here, we further characterized the sorption behavior of the novel composites (curcumin multiacrylate MNMs (CMA MNMs), quercetin multiacrylate MNMs (QMA MNMs), and polyethylene glycol MNMs (PEG MNMs)) by studying the effect of solution ionic strength, pH, and water hardness on their sorption capacities in order to establish an optimal working range for the application of the MNM gels as sorbents in environmental waters.

## 2. Results and Discussion

**Figure 1:**
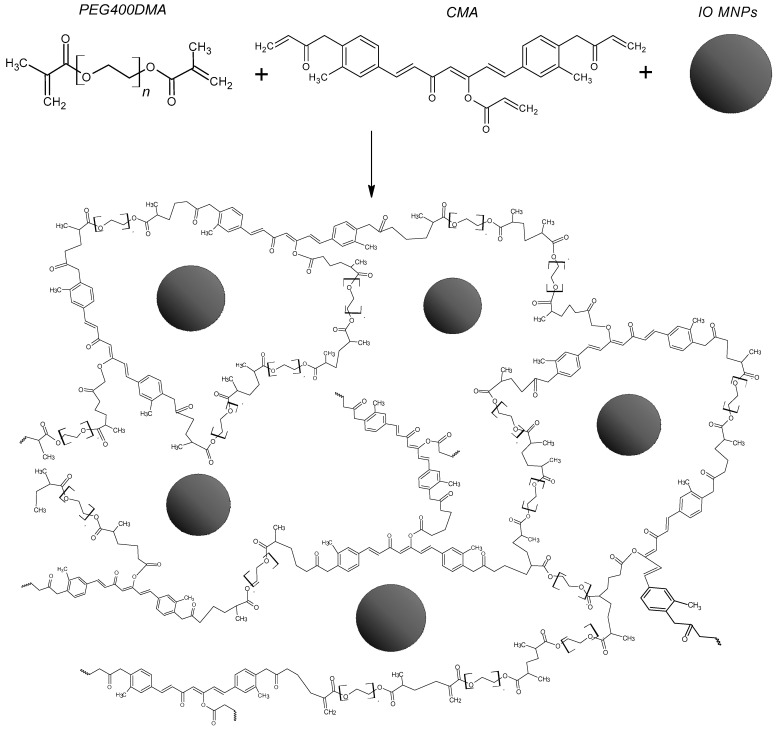
Schematic representation of the crosslinked polymer matrix interaction with the iron oxide magnetic nanoparticles within the magnetic nanocomposite microparticles (MNMs). Shown here are the CMA MNM gels for representation purposes. Here, the squiggly line represents the continuation of the polymer chain.

Magnetic nanocomposite microparticle (MNM) gels were prepared through chemically initiated free radical polymerization to obtain PEG-based crosslinked polymers with functional monomers from acrylated plant-derived polyphenols (CMA and QMA) with embedded magnetic iron oxide nanoparticles. The MNM gel systems were characterized as described in previous by from our group [22]. Briefly, Fourier transform infrared spectroscopy (FTIR) successfully confirmed the incorporation of the polyphenolic groups into the polymeric matrix used to enhance the binding affinity for PCB. The MNM gel systems obtained possessed an approximate 90:10 polymer network to magnetic nanoparticle composition, as determined through thermogravimetric analysis (TGA), which proved to be sufficient to maintain the magnetic properties of the microcomposite in order to use magnetic decantation as a separation technique after their application as sorbents [22]. Dynamic light scattering was used to determine the average hydrodynamic particle size for the MNM gels in a DI water solution, which ranged from 15 µm to 20 µm [22]. A schematic representation of the crosslinked polymer matrix interaction with the iron oxide magnetic nanoparticles within the MNM gels is depicted in Figure 1.

### 2.1. Effect of Solution Ionic Strength

**Figure 2:**
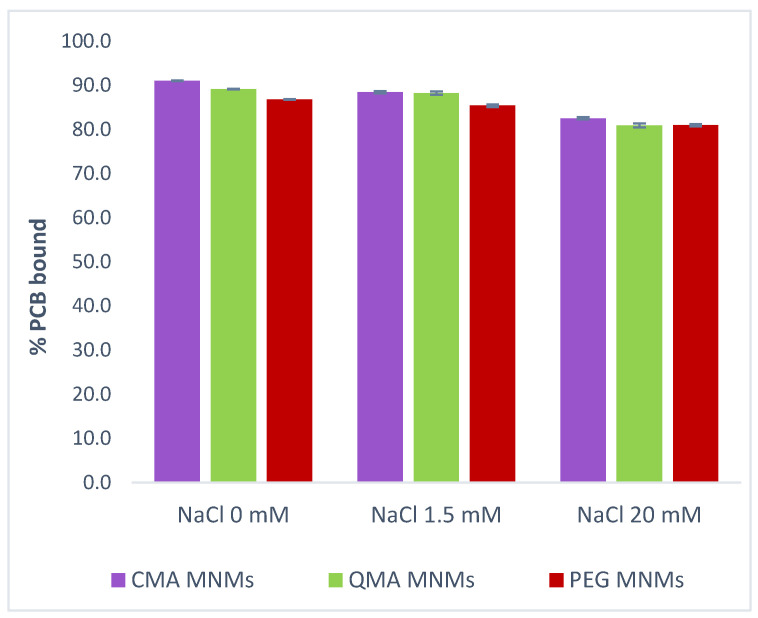
Effect of ionic strength on the sorption efficiency of PCB 126 on CMA MNMs, QMA MNMs, and PEG MNMs. The ionic strength concentrations represent fresh water (0 mM), surface water (1.5 mM), and ground water (120 mM).

The ionic strength of the solution is an important factor to study given the effect it can have on both the sorbent and the sorbate. In environmental water bodies, different ions will be present, of which sodium tends to be the most common. These ions can interact with the surface of the sorbate through electrostatic interactions and, potentially, weaken the sorption capacity of the sorbent material [25]. The effect of ionic strength on the sorption capacity of the MNM gel systems using sodium chloride as the model electrolyte is shown in Figure 2. The ionic strengths studied represent salinity levels of freshwater, surface water, and ground water (0, 1.5, and 20 mM, respectively) [37]. The general trend observed indicates that the effect of increasing the NaCl concentration does not appear to affect the binding capacity of the MNM gels. However, upon closer examination, when the ionic strength increases from 1.5 mM to 20 mM, there is a slight decrease in the binding capacity for all three MNM gel systems from 88% (CMA MNMs and QMA MNMs) and 86% (PEG MNMs) to ~82% and 81%, respectively. Similar behavior has been observed for PCB 126 adsorption to silicone rubber sorbent, wherein the sorption properties of the polymer decreased with increasing ionic strength of solution [21,38,39,40,41]. A different study conducted by Zhang et al. [42] demonstrated that the effect of sodium chloride salt in the range of 0 to 20 g/L^3^ had a minor effect on the PCB extraction of a phenyl functionalized fiber employed for solid-phase microextraction. Perez et al. [43] also observed an insignificant effect of sodium chloride concentration on the adsorption efficiency for PCBs of magnetic iron oxide nanoparticles. The overall decrease in PCB 126 binding of the MNM gel systems in this study is less than 4%, which correlates with what has been seen in the literature, and therefore it is possible to indicate that the ionic strength has a negligible effect on the sorption capacity of the developed MNM gels in the range studied.

### 2.2. Effect of Water Hardness

**Figure 3:**
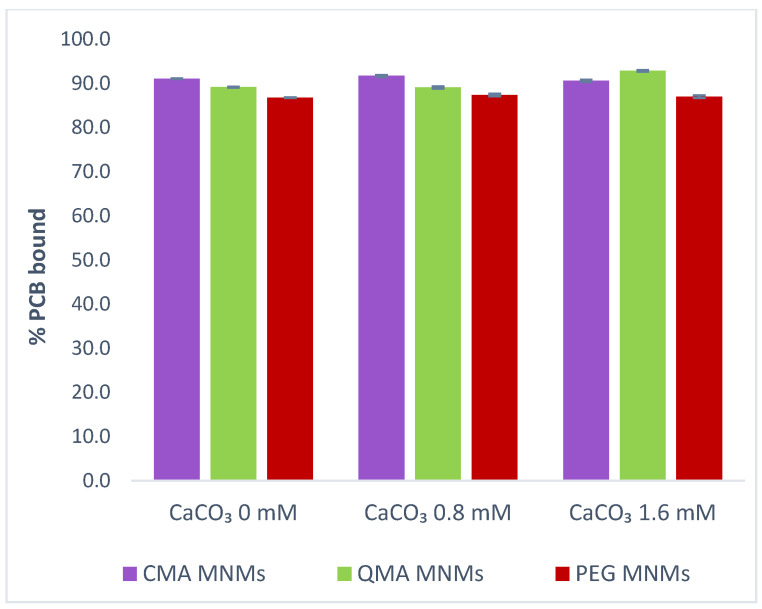
Effect of water hardness on the sorption efficiency of PCB 126 on CMA MNMs, QMA MNMs, and PEG MNMs. The water hardness concentrations represent soft (0 mM), moderately hard (0.8 mM), and hard (1.6 mM) waters.

Water hardness refers, mainly, to the amount of dissolved calcium and magnesium ions in water. The concentration of these ions varies depending on geographical location since it depends on the mineral composition of the rock and soils in the area. General guidelines for classifying water hardness are defined in terms of calcium carbonate concentration with waters ranging from 0 to 60 mg L^−1^ classified as soft, 61 to 120 mg L^−1^ classified as moderately hard, 121 to 180 mg L^−1^ classified as hard, and those with a concentration higher than 180 mg L^−1^ classified as very hard [44]. Given that very hard waters tend to be localized in regions with alkaline earths, the experimental conditions studied did not focus on calcium concentrations over 180 mg L^−1^. Figure 3 shows the effect of different water hardness conditions (soft, moderately hard, and hard), on the percent of PCB bound by the MNM gel systems. There appears to be no obvious effect on the binding capacity of the MNM gel systems caused by changes in water hardness.

### 2.3. Effect of Solution pH

**Figure 4:**
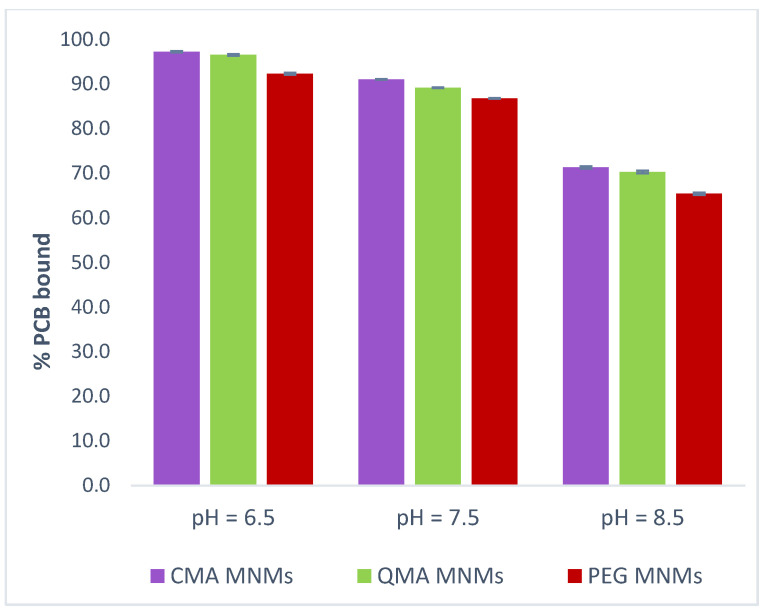
Effect of pH on the sorption efficiency of PCB 126 on CMA MNMs, QMA MNMs, and PEG MNMs.

The effect of pH on the sorption efficiency of a sorbent is one of the most important factors to evaluate. Changes in solution pH can alter the existing form of the sorbate of interest as well as the surface functional groups and density charges of the sorption sites on the sorbent. The pH of surface water has been described to range between 6.5 and 8.5, and the pH for shallow groundwater, from 6 to 8.5 [45]. Therefore, the pH studied was selected to be in this range. The effect of the different solution pH on the binding capacity of the MNM gel systems for PCB 126 is shown in Figure 4. The general trend observed is a decrease in the amount of PCB bound as the pH increases. Since PCB 126 is a neutral molecule and chemically stable under normal conditions, it is highly unlikely to be affected by changes in pH. This behavior corresponds to what has been reported in other studies [46,47]. Adeyinka and Moodley [34] conducted a study to determine the sorption of eight different PCBs onto differently sized soil particles showed a decrease in binding as the pH increased, with optimal sorption occurring between pH 6.5 and 7.5. Similarly, Taha and Mobasser [48] noted that the adsorption of multiwall carbon nanotubes (MWNT) and nano-clay for PCBs decreased as the pH increased from 3 to 10, with the maximum adsorption occurring at around pH 7, explaining that, for carbonaceous materials, generally, an increase in pH leads to increased ionization, solubility, and hydrophilicity of sorbent, which decreases adsorption for organic compounds. Moreover, Choi et al. [49] have also reported the removal of almost all studied PCBs using a nano-Fe/Pd bimetallic system-impregnated activated carbon to occur at pH 6.5. 

To adjust the pH of the 99:1 DI water to ethanol solvent (where the binding studies were being carried out) to 6.5, a phosphate citrate buffer was used. Here, the amount of PCB bound appears to slightly increase when compared to the standard binding conditions at a pH of 7.5. When the pH decreased from 7.5 to 6.5, the average increase in capacity for all the MNM gels was around 6%. This result is in agreement with previously published data showing that a pH between 6.5 and 7.5 was optimal for maximum adsorption of eight PCB congeners to occur [34,46,47,48,49].

On the other hand, to adjust the pH to 8.5, a glycine sodium hydroxide buffer was used. Under these conditions, it was seen that as the pH increased from 7.5 to 8.5, the amount of PCB bound decreased from 91 to 71% for the CMA MNMs, 89 to 70% for the QMA MNMs, and 86 to 65% for the PEG MNMs. Here, the average decrease in capacity for all the MNM gels was almost 20%. This significant decrease in binding capacity correlates to the general effect of ionic strength observed for the MNM gel systems. This drastic decrease in PCB binding in alkaline solutions has also been reported by Lv et al. [50] for biomass-derived porous materials employed in water.

Because two different buffer solutions were employed in this study, it is reasonable to consider the combined effect of pH and the presence of ions in solution to explain the observed results. The main mechanism of interaction between the PCB 126 molecules and the surface of the MNM gels is attributed to π-π interactions between the aromatic rings present in both the sorbate and the sorbent. π-π stacking interactions have been widely documented as the mechanism enabling PCB binding with a variety of sorbents [32,33,34,46,48,50,51,52]. Additionally, the presence of anions in solution increases with increasing pH, allowing a different type of noncovalent interaction to occur: anion-π interactions. These kinds of interactions are usually defined as attractive interactions between anions and the faces of π-rings [53,54]. Specifically, π-π electron coupling interactions occur between the π electrons on the surface of the sorbent and the Cl^−^ π electrons present in the PCB molecules [48,55]. In the present work, anion-π interactions can also occur between the aromatic rings of the PCB 126 molecules and the anions from the buffers, resulting in an increase in the solubility of the PCBs in solution, thus decreasing in their sorption onto the MNM gel system. Similarly, anion-π interactions also occur between the aromatic rings within the MNM gel network, creating a competing effect with PCB 126 for the binding sites in the sorbent. Even though anion-π interactions are said to be weaker than π-π interactions, they can sometimes form “complexes” in solution, which can suppress their sorption. Anion-π is a relatively newer type of noncovalent interaction and, as such, there is still not enough information available to make conclusive comments on this.

Although different buffer solutions were used in this study, it is anticipated that the MNM gel systems will be affected by the pH range, specifically at higher pHs, due to possible ionization of the functional groups on their surfaces. Overall, the binding affinity of the MNM gel systems for PCB 126 decreased as the pH increased from 6.5 to 8.5, given the multiplicity of π-π and π-anion interactions occurring in solution between the PCB molecules and the sorbent and the PCB molecules and the buffer anions. These results indicate that solution pH is an important factor to consider in the application of MNM gel systems as sorbents in natural bodies of water, and its control will be necessary.

## 3. Conclusions

A water body is a complex system, and there are many factors that can influence the behavior of sorbents used in water remediation. Here, the effects of ionic strength, water hardness, and solution pH on the sorption capacity of PCB 126 were evaluated for three novel magnetic nanocomposite microparticles (CMA MNMs, QMA MNMs, and PEG MNMs gels) previously synthesized by our group. The overall aim of these studies was to establish an optimal working range for the application of the MNM gels as sorbents in environmental waters. The results showed that ionic strength and water hardness had little to no impact on the sorption capacity of the MNM gel systems towards PCB 126, in the range studied, suggesting the MNM gels can be used in a wide range of applications without the need to control these factors. However, the solution pH was seen to affect the binding capacity of the MNM gels for PCB 126, resulting in decreased binding as the pH increased from 6.5 to 8.5. The decrease in binding with increasing pH can be attributed to -π interactions occurring between the aromatic rings of PCB 126 and the anions from the buffers, resulting in an increase in the solubility of the PCBs in solution, thus hindering their sorption. These results indicate that the developed MNM gels, with high affinity for PCB molecules and their inherent magnetic separation capability, show promising potential for their application as magnetic sorbents for polychlorinated biphenyls in groundwater and surface water remediation, provided that solution pH is controlled.

## 4. Materials and Methods

### 4.1. Materials

Iron (III) chloride hexahydrate (FeCl_3_·6 H_2_O); iron chloride tetrahydrate (FeCl_2_·4 H_2_O; ammonium persulfate (APS); N,N,N′-Trimethylethylenediamine 97% (TEMED); triethyl amine (TEA); acryloyl chloride; potassium carbonate (K_2_CO_3_); dibasic sodium phosphate (Na_2_HPO4); and glycine (C_2_H_5_NO_2_) were obtained from Sigma Aldrich (St. Louis, MO, USA). Ammonium hydroxide (NH_4_OH) was purchased from EMD Chemicals (Gibbstown, NJ, USA). Poly(ethylene glycol) 400 dimethacrylate (PEG400DMA) was obtained from Polysciences INC. (Warrington, PA, USA). Curcumin was purchased from Chem-Impex International, Inc. (Bensenville, IL, USA), and quercetin was purchased from Cayman Chemicals (Ann Arbor, MI, USA). Citric acid monohydrate, sodium chloride (NaCl), calcium carbonate (CaCO_3_), and sodium hydroxide (NaOH) were obtained from Fisher Scientific (Hannover Park, IL, USA). 3,3′,4,4′,5-Pentachlorobiphenyl (PCB-126) in isooctane was purchased from Accustandard (New Haven, CT, USA). 5′-fluoro-3,3′,4,4′,5-pentachlorobiphenyl (F-PCB 126) was purchased from Resolution Systems Inc. (Holland, MI, USA). All solvents (Isooctane, ethanol HPLC grade, tetrahydrofuran (THF), dichloromethane (DCM), acetonitrile (ACN), and acetone) were obtained from Fisher Scientific (Hannover Park, IL, USA). All materials were used as received.

### 4.2. Magnetic Nanocomposite Microparticle Synthesis

Magnetic nanocomposite microparticles (MNMs) were synthesized via chemically initiated free radical polymerization followed by cryomilling, as previously described by our group [9]. Poly-(ethylene glycol) 400 dimethacrylate (PEG) and an acrylated polyphenol were reacted in DMSO using ammonium persulfate dissolved in ethanol as the initiator for the reaction and N,N,N′,N′-tetramethylethylenediamine as the accelerator to create a crosslinked polymer network. Iron oxide nanoparticles (IO MNPs) were added as the reaction took place, resulting in their immobilization within the polymer matrix.

### 4.3. Particle Characterization

The magnetic nanocomposite microparticles were characterized as described in previous work by our group [22]. Fourier transform infrared spectroscopy (FTIR) was conducted to validate the incorporation of the polyphenolic groups into the polymer matrix. Thermogravimetric analysis (TGA) of the microcomposites was run to quantify the percentage of mass corresponding to the magnetic nanoparticles within the MNM gel system. Dynamic light scattering (DLS) measurements of microparticle suspension in DI water were conducted to determine the hydrodynamic sizes of the MNM gels. To determine the stability of the microparticle systems in a DI water suspension, UV-visible spectroscopy was utilized to record their change of absorbance over a 12 h period. 

### 4.4. PCB Binding Studies

Binding studies were conducted at equilibrium conditions. All samples were prepared by weighing 0.1 mg of the dry MNM gels into 3 mL borosilicate glass vials and dispersing them in DI water. The MNM gel systems studied were CMA MNM, QMA MNM, and PEG MNM gels. Due to the low solubility of PCB 126 in water, a concentrated stock solution was prepared in ethanol, which was then used to spike each sample to obtain the desired concentration of 0.05 ppm. Samples were placed in an orbital shaker for 48 h at room temperature. Following this, the samples were magnetically separated using a static magnet for approximately 20 min in order to guarantee that all MNM gels were decanted from solution. The supernatant containing the free PCB 126 was collected and transferred to a new vial, where a liquid extraction using isooctane was performed for 24 h. Finally, the organic phase was transferred into a glass chromatography vial using a Hamilton syringe and spiked with a known amount of the internal standard, 5′-fluoro-3,3′,4,4′,5-pentachlorobiphenyl (F-PCB 126). The PCB 126 present in each sample was determined using an Agilent 6890 N gas chromatograph coupled with electron capture detection (CG-ECD) equipped with an Agilent HP-5MS UI column (30 × 0.25 × 0.25).

The amount of PCB bound to the MNM gel systems was calculated as

(1) $$\%\;Bound=\frac{C_{o}-C_{e}}{C_{o}}\times 100,$$

where $C_{o}$ (mg L^−1^) is the initial concentration of PCB 126, and $C_{e}$ (mg L^−1^) is the concentration of PCB 126 at equilibrium. 

The influence of ionic strength, water hardness, and pH on the sorption efficiency of the MNM gels was investigated. The influence of ionic strength was tested using NaCl at two different concentrations: 1.5 mM (or mol/m^3^) and 20 mM (or mol/m^3^). The effect of water hardness was evaluated using CaCO_3_ at two different concentrations: 0.8 mM and 1.6 mM. The pH of the solution was adjusted to 6.5 using a glycine-NaOH buffer, and to 8.5 using a phosphate-citrate buffer, in order to assess its effect on PCB 126 binding.

## Data Availability

The data presented in this study are available upon request from the corresponding author.
